# Future science prize goes to EMI editorial board members and the science of emerging infections

**DOI:** 10.1080/22221751.2021.2020599

**Published:** 2022-01-16

**Authors:** Shan Lu, Yu-mei Wen

**Affiliations:** Editorial Office, Emerging Microbes & Infections


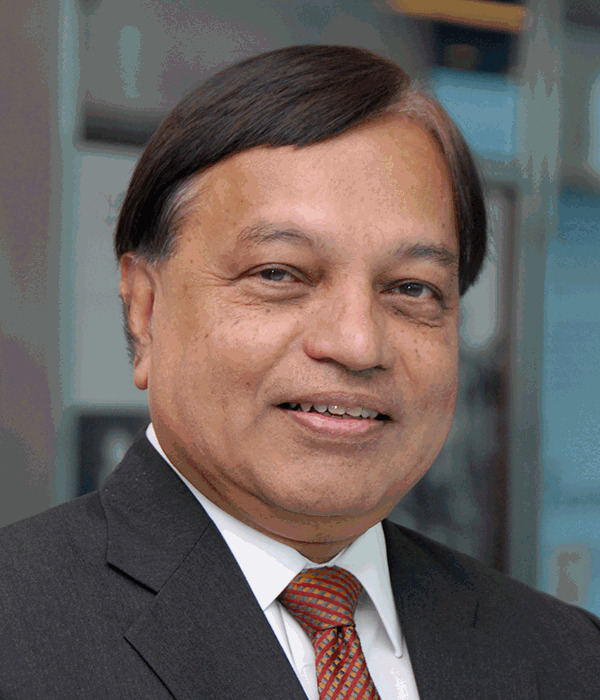
 
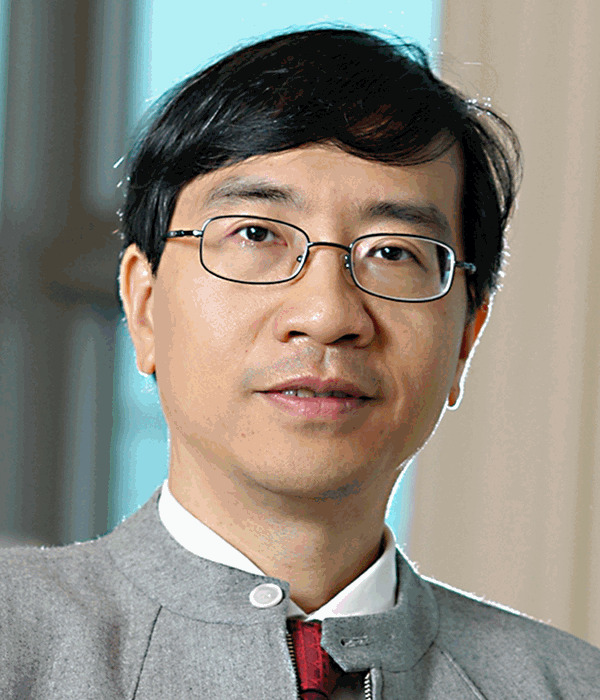


2021 Future Science Prize winners were announced recently and two editorial board members of EMI, Professor Kwok-Yung Yuen and Professor Joseph Sriyal Malik Peiris, both from Hong Kong University, won the Prize in Life Science for “their discoveries of SARS-CoV-1 as the causative agent for the global SARS outbreak in 2003 and its zoonotic origin, with impact on combating Covid-19 and emerging infectious diseases.”

During the outbreak of severe acute respiratory syndrome (SARS) in 2003, Prof Yuen, Prof Peiris, and their team were consulted on the first SARS patients in Hong Kong and isolated SARS-CoV-1 from their clinical specimens, which was critical to the design of diagnostic tests and disease characterization [1]. In addition, Prof Yuen’s continued studies on SARS-like viruses in wild bats greatly increased our knowledge of zoonotic reservoirs, barriers to cross-species transmission, pathogenesis, and laboratory diagnosis of these viruses. Because of the high prevalence of SARS-like coronavirus in bats, the discovery predicted the potential re-emergence of a SARS-like epidemic and stressed the importance of public health preparedness.

Prof Kwok-Yung Yuen, 65 years old and a Hong Kong native, obtained his MD from the University of Hong Kong in 1998. He is a clinical specialist in microbiology with qualifications of MBBS (HK), MD (HK), Fellow of the Royal College of Physicians (FRCP, Lond, Edin), Surgeons (FRCS, Glas), and Pathologists (FRCPath, UK). He is a member of both the Chinese Academy of Engineering (Division of Medicine & Health) and the Chinese Academy of Medical Sciences, and a Fellow of the American Academy of Microbiology.

During the outbreak of SARS in 2003, he led his team in the discovery of the SARS coronavirus. Subsequently, he discovered the natural reservoir of bat SARS-related coronaviruses, which are predecessors of human SARS-CoV-1 of 2003 and SARS-CoV-2 of 2021, in Chinese horseshoe bats. His findings renewed the interest of bats as the source of novel microbes causing emerging infectious diseases. The success in finding novel microbes in human and animals is exemplified by his other discoveries of human coronavirus HKU1(still circulating globally as common cold coronavirus), bat coronaviruses HKU2 (related to farm outbreaks of porcine diarrheal disease), HKU4 and HKU5 (related to MERS coronavirus), and HKU15 (related to the suspected porcine delta coronavirus jumping into human in 2021 which is also closely related to the Sparrow coronavirus HKU17). Their team has discovered a total of 35 coronaviruses.

During the COVID-19 pandemic, Prof Yuen and his team were the first to report in The Lancet on the identification of the first familial cluster of COVID-19, thus confirming person-to-person transmission of SARS-CoV-2, which led to major policy changes for controlling the pandemic worldwide.

Prof Yuen is currently the Chair of Infectious Diseases of the Department of Microbiology at the University of Hong Kong. He is also the Co-Director of the State Key Laboratory of Emerging Infectious Diseases. His publications on emerging infectious diseases and microbial hunting have >90,000 citations and h-index of 121 (Scopus). He was ranked by Clarivate Analytics as one of the world’s most highly cited researchers and top 1% scholars.

Prof Joseph Sriyal Malik Peiris, born in 1949 in Sri Lanka, obtained his PhD from the University of Oxford in 1981, is currently a professor at the University of Hong Kong. Professor Malik Peiris had his undergraduate medical education in Ceylon (now Sri Lanka), followed by postgraduate training in virology at the University of Oxford, UK. He joined The University of Hong Kong in 1995, and is now Chair of Virology at the School of Public Health, The University of Hong Kong. He co-directs the WHO H5 Reference Laboratory and the WHO SARS-CoV-2 reference laboratory at The University of Hong Kong. He also serves on many Hong Kong and WHO advisory committees, including the WHO International Health Regulations Emergency Committee on COVID-19.

He is a clinical and public health virologist with a particular interest in emerging viral diseases at the animal–human interface – using the “One-Health” approach. These include human and animal influenza viruses such as avian influenza subtypes H5N1 and H7N9, the emergence of the 2009 pandemic H1N1 from swine, and coronaviruses such as SARS-CoV-1, MERS-CoV, and SARS-CoV-2.

In 2003, Professor Peiris and his research team discovered SARS-CoV, a novel coronavirus, as the etiological agent for SARS. He has contributed to understanding the emergence, spread and pathogenesis of SARS, and of viral zoonotic avian influenza diseases, including their control. He has served on WHO missions to Saudi Arabia on MERS and to China on avian influenza H7N9. He has close collaborations with Professor Zhong Nanshan and his team at the State Key Laboratory of Respiratory Disease in Guangzhou, China as well as other international peers. His research team is currently investigating the recently emerged SARS-CoV-2 that caused the COVID-19 pandemic.

Professor Peiris has over 800 peer reviewed research publications in international scientific journals, which have been cited over 76,000 times. In recognition of his research contributions, he was elected as a Fellow of the Royal Society of London (2006). He has received the Mahathir Science Award, Akademi Sains, Malaysia (2007); a Silver Bauhinia Star, Hong Kong SAR (2008); the Medal “100 Years of Virology” of the D.I. Ivanovsky Institute of Virology of the Russian Academy of Medical Sciences (2009); and the title of Officier de la Legion d’Honneur, Republic of France (2017). He was elected a Foreign Associate of National Academy of Sciences, USA (2017), and awarded the John Dirks Canada Gairdner Global Health Award (2021).

Prof Yuen is a founding member of EMI Editorial Board since 2012, and is a current Associate Editor of EMI. Prof Peiris joined EMI Editorial Board in 2019. Both have provided significant contribution and guidance to the rapid growth of EMI over the last 10 years.

The recognition of Kwok-Yung Yuen and Joseph Sriyal Malik Peiris and their work by this year’s Future Science Prize not only highlighted their individual seminal contributions to science but also reminded everyone the heavy impact of emerging infectious diseases on human society as we are still learning from the ongoing COVID-19 pandemic. EMI is committed to continue our mission in transmitting the highest quality of scientific research work to the global and we are extremely proud to have colleagues like Profs Yuen and Peiris among our editorial board.

## References

[1] Peiris JSM, Lai ST, Poon LLM, et al. Coronavirus as a possible cause of severe acute respiratory syndrome. The Lancet. 2003 Apr. doi:10.1016/S0140-6736(03)13077-2 PMC7112372 12711465

